# Flavonoid Fisetin Alleviates Ovarian Aging of Laying Chickens by Enhancing Antioxidant Capacity and Glucose Metabolic Homeostasis

**DOI:** 10.3390/antiox13121432

**Published:** 2024-11-21

**Authors:** Zhaoyu Yang, Jiaxuan Zhang, Qiongyu Yuan, Xinyu Wang, Weidong Zeng, Yuling Mi, Caiqiao Zhang

**Affiliations:** Department of Veterinary Medicine, College of Animal Sciences, Zhejiang University, Hangzhou 310058, China; 12117050@zju.edu.cn (Z.Y.); 3200101330@zju.edu.cn (J.Z.); qiongyu@zju.edu.cn (Q.Y.); 22217098@zju.edu.cn (X.W.); zengwd@zju.edu.cn (W.Z.)

**Keywords:** fisetin, oxidative stress, glucose catabolism, ovarian aging, chicken

## Abstract

Oxidative stress is a crucial factor contributing to ovarian follicular atresia and an imbalance in ovarian energy metabolism in poultry, leading to decreased laying performance in aging hens. This study aimed to investigate the effects of a natural flavonoid, fisetin, on laying performance, ovarian redox status, and energy metabolism in laying chickens. The results showed that dietary fisetin supplementation improved egg production and eggshell quality in aging laying chickens, reduced follicular atresia rate, promoted ovarian cell proliferation, elevated serum estrogen and progesterone levels, restored ovarian antioxidant capacity, and improved energy metabolism. Furthermore, fisetin treatment increased the activity of antioxidant enzymes by inhibiting NF-κB signaling and COX-2 expression while promoting SIRT1 expression in the H_2_O_2_-induced small white follicle (SWF). Additionally, fisetin significantly enhanced the anti-apoptotic capacity of SWF and promoted glucose catabolism by activating the AKT and JNK signaling pathways. In summary, fisetin supplementation can alleviate ovarian oxidative stress in aging laying chickens by upregulating SIRT1 expression and inhibiting NF-κB signaling. The activation of AKT and JNK signaling pathways by fisetin contributes to the balance of energy metabolism and promotion of follicular development in the ovaries of aging laying chickens, thereby retarding ovarian aging in poultry production.

## 1. Introduction

Ovarian follicle depletion and endocrine dysfunction result in the loss of female fertility, known as ovarian aging [1]. Reproductive aging is primarily due to an age-related decline in both the quantity and quality of ovarian follicles. Since ovarian aging occurs earlier than aging in other organs and tissues in women, and its effects extend beyond the reproductive system—indirectly leading to conditions such as osteoporosis, cardiovascular disease, and obesity—studying ovarian aging is both critical and urgent [2]. Similarly, the ovarian quality of laying hens diminishes over time, especially in hens with high laying performance after genetic breeding. High-yielding chickens typically reach peak egg production around day 280, but after this peak, egg production gradually declines, and by 580 days, they rarely lay eggs [3]. These changes in laying hen productivity cause significant economic losses to the poultry industry. Therefore, addressing the prolongation of the laying cycle and mitigating ovarian aging in poultry is imperative.

The ovary is a highly dynamic and heterogeneous organ, composed of various cell types, including follicles at different developmental stages (primordial, primary, secondary, and antral follicles) [4]. It houses the entire pool of female oocytes from birth, with over 99% of follicles undergoing natural atresia during the transition from the preantral to the antral stage [5]. Disturbances in the ovarian microenvironment, such as DNA damage, reactive oxygen species (ROS) overload, and endoplasmic reticulum stress (ERS), cause oxidative damage to cells, which in turn triggers apoptosis [6,7]. This process is closely linked to ovarian aging, which is characterized by accelerated and abnormal follicular atresia, primarily due to granulosa cell apoptosis [8,9]. In the ovaries of laying hens, excessive ERS induces apoptosis in granulosa cells, accelerating follicular atresia [10]. Additionally, even slight disturbances in the follicular fluid microenvironment can negatively affect energy metabolism and disrupt mitochondrial dynamics in both granulosa cells and oocytes. These disturbances contribute to accelerating ovarian aging [11]. Our previous research showed that glycolysis rates in granulosa cells from the ovaries of aged laying hens were significantly lower than those in young hens [12]. Overall, ovarian aging is closely associated with an imbalance in the redox system and abnormalities in energy metabolism.

Natural antioxidants have been found to exhibit significant effects in improving reproductive performance in poultry [13,14]. Fisetin (3,3′,4′,7-tetrahydroxy flavone), a natural flavonoid with potent antioxidant, anticancer, and anti-inflammatory properties, is found in various fruits and vegetables, including strawberries, apples, persimmons, onions, and cucumbers [15]. It is recognized as a prominent senolytic agent that promotes the elimination of senescent cells and diminishes the production of senescence-associated secretory phenotypes [16]. Furthermore, fisetin exerts anti-ferroptotic and anti-inflammatory effects, as it inhibits the release of pro-inflammatory cytokines (TNFα, IL-6) and regulates the expression of ferroptosis markers (ACSL4, COX2, HMGB1) [17,18]. It effectively scavenges ROS, reducing oxidative stress-induced cellular damage. Research indicates that intermittent supplementation of fisetin (100 mg/kg/day) in aged mice lowers ROS levels in the aortic mitochondria by inhibiting the expression of mitochondrial NADPH oxidase and enhancing the expression of antioxidant enzymes like MnSOD [19]. Moreover, fisetin delays postovulatory oocyte aging by enhancing SIRT1 activity, increasing GSH level and *Sod2* transcription level [20]. Numerous investigations have highlighted the potential of fisetin as a novel antioxidant. However, no research has been conducted on the effects of fisetin on reproductive performance in poultry.

Given fisetin’s crucial role in counteracting cell apoptosis, we hypothesized that fisetin provides protection against oxidative damage in both a “natural aging” model and “chemotherapy-induced” model. We investigated the effects of fisetin on antioxidant capacity, cell cycle regulation, energy metabolism, and follicular development in the ovaries of laying chickens. This study aims to develop practical strategies to mitigate ovarian aging, thereby extending the laying period of chickens.

## 2. Materials and Methods

### 2.1. Animal and Sample Collection

Hyline White laying chickens (*Gallus domesticus*) were obtained from a local commercial farm (Huajie Poultry Company, Hangzhou, China). In an in vivo feeding trial lasting 21 days, thirty laying chickens, aged 580 days (D580) and weighing between 1.5–2 kg, were randomly divided into two groups: a control group and a fisetin feeding group, with 15 hens in each group (five hens/cage across three cages). The trial was conducted at a temperature range of 28–32 °C under controlled environmental conditions. All chickens were provided with a diet and drinking water based on their nutritional requirements. The treatment group received fisetin at a dosage of 50 mg/kg/day (MB5836-1, MeilunBio, Dalian, China), mixed with water to form a suspension for gavage. The total phenol content of fisetin used in the study was calculated by the Total Phenols Colorimetric Assay Kit (E-BC-K354-M, Elabscience, Wuhan, China) and found to be 87.88 ± 0.65 mg/g. The total phenol intake per hen was calculated to be 6.59–8.79 mg based on 1.5–2 kg B.W. per hen. The control group received 1 mL of pure water. Egg production was recorded daily for each group. On the last day of the feeding trial, blood samples were collected from the wing veins of each laying chicken prior to sacrifice. Ovarian tissue, comprising small white follicles (SWF, φ2–4 mm) and cortical portions, was aseptically extracted from each hen using scissors for further analysis.

### 2.2. Culture of Ovarian Tissue and Chemical Treatments

Briefly, SWF or ovarian tissues were maintained under constant conditions at 38.5 °C with 5% CO_2_ using DMEM culture medium (HyClone, Logan, UT, USA) supplemented with 100 IU/mL penicillin, 100 μg/mL streptomycin (HyClone, Logan, UT, USA), and 5% fetal bovine serum (Hyclone, Logan, UT, USA). The NF-κB inhibitor JSH-23 and the AKT inhibitor AT7867 (MedChemExpress, Monmouth Junction, NJ, USA) were used as indicated, with SWF or ovarian tissue treated with 50 μM JSH-23 and 100 μM AT7867, respectively. The culture medium and treatments were refreshed every 24 h, with the addition of 10 μg/mL 5-bromo-2′-deoxyuridine (BrdU, Sigma-Aldrich, St. Louis, MO, USA) during the final 24 h. SWFs obtained from 280-day-old (D280) peak laying chickens were exposed to varying concentrations of fisetin (1, 10, 100 μM) for 72 h, followed by co-culturing with H_2_O_2_ for the final 24 h in vitro. Furthermore, SWFs from D580 hens were co-cultured with 100 μM fisetin for 72 h in vitro. After the culture period, samples were collected for further analysis.

### 2.3. Morphological Analysis

Ovaries and SWF were collected and fixed in 4% paraformaldehyde for 24 h, followed by paraffin embedding and serial sectioning at 5 μm thickness. Hematoxylin and eosin (H&E) staining was then performed according to standard protocols. Microscopic imaging of the stained sections was conducted using an Eclipse 80i microscope (Nikon, Tokyo, Japan).

For immunohistochemistry (IHC), isolated ovaries and SWF were fixed and permeabilized with 4% PFA prior to paraffin sectioning. After deparaffinization and rehydration, sections were treated with 3% H_2_O_2_ to block endogenous peroxidase activity. After antigen retrieval, sections were blocked with goat serum for 1 h at room temperature, then incubated with primary antibodies overnight at 4 °C. After PBS washes, horseradish peroxidase-conjugated secondary antibodies were applied and incubated at 37 °C for 1 h. Visualization was achieved by DAB staining, and hematoxylin was used for nuclear counterstaining. Imaging of IHC-stained samples was conducted using a microscope (Nikon, Tokyo, Japan). Immunofluorescence (IF) staining followed a protocol similar to IHC, with additional goat serum blocking prior to applying primary antibodies, including anti-PCNA (Abcam, Cambridge, UK) and anti-BrdU (G3G4, DSHB, Iowa City, IA, USA). After overnight incubation with primary antibodies and subsequent PBS washing, samples were incubated with Alexa Fluor 488 and TRITC-conjugated secondary antibodies for 1 h at 37 °C. Imaging of IF-stained samples was performed using a spinning disk super-resolution confocal microscope (SpinSR, Olympus, Tokyo, Japan).

### 2.4. TUNEL Assay

TUNEL-positive apoptotic cells in SWF were identified in paraffin sections using a TUNEL apoptosis assay kit (Vazyme, Nanjing, China) according to the manufacturer’s instructions. The sections were then subjected to DAPI staining for 10 min and observed with an IX70 fluorescence microscope (Olympus, Tokyo, Japan). Three random fields were analyzed per slide, with five SWF per animal. ImageJ v1.44 software was used to quantify the TUNEL-positive cell rate in SWF.

### 2.5. Western Blotting

SWF was lysed using RIPA buffer (MeilunBio, Dalian, China) supplemented with protease inhibitor (MB2678, MeilunBio) and phosphatase inhibitor (Solarbio, Beijing, China) to extract total protein. Protein quantification was performed using a BCA kit (Nanjing Jiancheng Institute of Bioengineering, Nanjing, China). Proteins were separated on 10% SDS-PAGE gels and then transferred to a polyvinylidene-fluoride (PVDF) membrane. The membranes were first probed with primary antibodies (Table S1). Blots were visualized using a FDbio-Femto ECL Substrate Kit (Fdbio, Hangzhou, China), and signals were captured using the ChemiDoc Touch gel imaging system (Bio-Rad, Hercules, CA, USA). ImageJ v1.44 software was used for image analysis and quantification, with normalization to β-actin as an internal control.

### 2.6. RNA Extraction and qRT-PCR

Total RNA extraction from SWF was performed using TRIzol reagent (Takara, Osaka, Japan) according to the manufacturer’s instructions. The isolated RNA was reverse transcribed into cDNA using the HiScript II 1st Strand cDNA Synthesis Kit (R211-02, Vazyme). Subsequently, qRT-PCR was conducted on a CFX96 Touch real-time PCR detection system (C1000 Touch, Bio-Rad, Hercules, CA, USA) using ChamQ Universal SYBR qPCR Master Mix (Q711-02, Vazyme). Normalization was performed using β-actin, and the relative mRNA expression of target genes was determined using the 2^−ΔΔCt^ method. The primers used in this study are listed in Table S2.

### 2.7. Measurement of Antioxidant Indices in SWF and Serum Reproductive Hormone Levels

SWF was homogenized in a chilled saline buffer, followed by centrifugation to collect the supernatants. The supernatants were used to assess the antioxidant capacity including measurements of glutathione S-transferase (GSH-ST), total antioxidant capacity (T-AOC), total superoxide dismutase (T-SOD), catalase (CAT), glutathione peroxidase (GSH-Px), glutathione (GSH), malonaldehyde (MDA), and H_2_O_2_ using commercially available reagent kits (Nanjing Jiancheng Institute of Bioengineering). Serum 17β-estradiol (E_2_) and progesterone (P_4_) levels were quantified by chemiluminescence assay at the medical laboratory of the Affiliated Hospital of Hangzhou Normal University (Hangzhou, China).

### 2.8. Determination of Egg Quality

Eggs were collected at fresh lay on the final day of the feeding trial and tested for quality within 12 h of collection. All samples were kept at room temperature prior to egg quality assessment. Egg weight, shell strength, egg white height, yolk color score, and Haugh unit value were measured using a digital egg tester (DET6000, NABEL Co., Ltd., Kyoto, Japan). Eggshells were scanned with a scanning electron microscope (TM-1000, Hitachi, Tokyo, Japan), including cross section, inner surface, and outer surface. Six individual egg samples were scanned for each experimental group.

### 2.9. Statistical Analysis

Significant differences were determined using unpaired Student’s *t*-tests and one-way ANOVA in GraphPad Prism 8.0 software. Values are presented as mean ± SEM, with statistical significance denoted by *p* < 0.05.

## 3. Results

### 3.1. Laying Performance and Egg Quality

The effect of fisetin on egg production and laying performance is presented in Table 1. Dietary supplementation with fisetin increased average egg weight, egg production, eggshell strength and thickness, and changed the egg yolk color compared with the Con group (*p* < 0.01). However, there was no influence on albumen height (*p* = 0.35) and Haugh unit (*p* = 0.179) after fisetin intake.

### 3.2. Fisetin Attenuated Ovarian Degradation in the NA-OF Model

**Figure 1:**
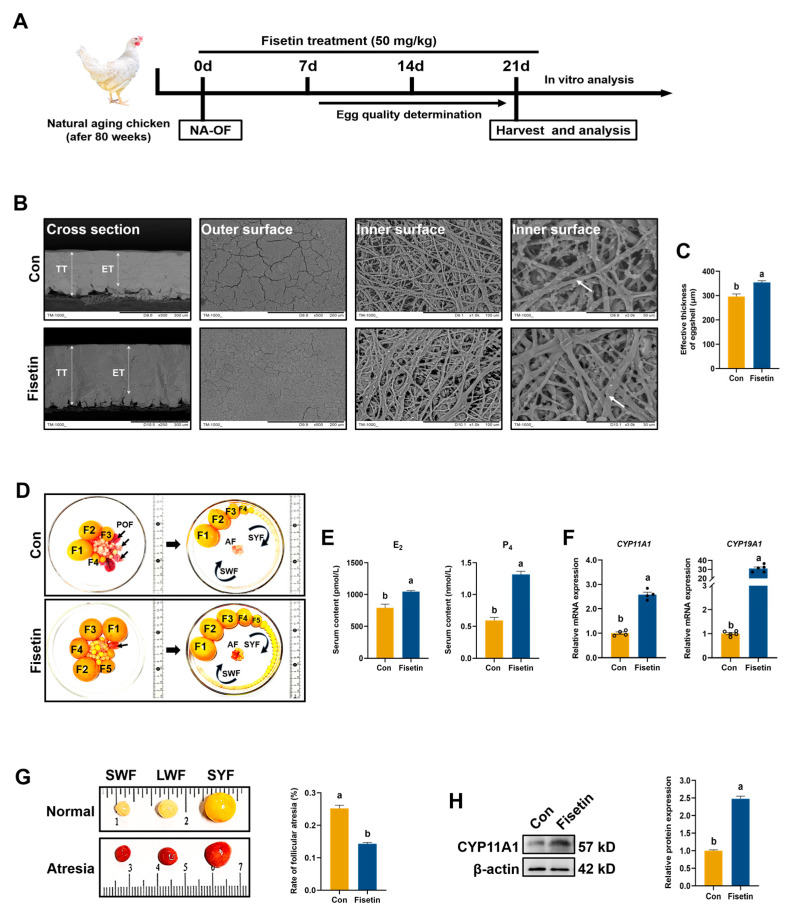
Effect of fisetin on follicle development, egg quality, and hormone balance in NA-OF model. (**A**) Experimental design of fisetin treatment and analysis in NA-OF model. (**B**) Scanning electron microscope (SEM) images of eggshells, displaying cross-sections, outer surfaces, and inner surfaces. TT: Total thickness; ET: effective thickness. White arrows indicate calcareous fibers. (**C**) Eggshell effective thickness (*n* = 6). (**D**) Ovaries of aged laying chickens (580-day-old). Avian ovarian follicles are generally categorized by size or color: large preovulatory follicles (F1, F2, F3, etc.), small yellow follicle (SYF), large white follicle (LWF), and small white follicle (SWF). Black arrow: post-ovulation follicle; AF: atretic follicle. (**E**) Levels of serum estrogen (E_2_) and progesterone (P_4_) (*n* = 15). (**F**) Relative mRNA expression of steroid synthesis-related genes (*CYP11A1*, *CYP19A1*) in SWF. (**G**) Images and rate of atretic follicles (*n* = 6). (**H**) Western blot and quantitative analysis of CYP11A1 expression in SWF (*n* = 3). Values are shown as mean ± SEM. Different letters represent statistically significant differences among the groups (*p* < 0.05).

We investigated the effect of fisetin on aged laying chickens using a natural aging ovarian follicle model (NA-OF) to mimic progressive ovarian degradation. The schematic timeline of the fisetin treatment is shown in Figure 1A. Initially, we assessed the toxicity of fisetin. None of the chickens died after 21 days of treatment. SEM images of eggshells revealed a denser and thicker fiber structure on the inner surface and more uniform outer surface cracks in the fisetin group (Figure 1B). Fisetin treatment also increased eggshell effective thickness compared with the control group (*p* < 0.001, Figure 1C). Additionally, fisetin supplementation increased the number of small yellow follicles (SYFs), potentially developing into reserve follicles for preovulatory follicles, compared with the control diet (Figure 1D). Treatment with fisetin normalized blood E_2_ and P_4_ levels (*p* < 0.001, Figure 1E). Furthermore, the mRNA levels of *CYP11A1* and *CYP19A1*, which are required for steroid hormone synthesis, was higher than the con group in the fisetin group (*p* < 0.001, Figure 1F), and the protein level of CYP11A1 was also elevated after fisetin intake (*p* < 0.001, Figure 1H). Moreover, fisetin reduced the rate of atretic follicles, which were characterized by increased vascularity and a soft, inelastic texture that hindered their development into preovulatory follicles (Figure 1G). Taken together, fisetin could halt multiple aspects of ovarian degradation in aged laying chickens.

### 3.3. Effect of Fisetin on Ovarian Cells and Antioxidant Capacity in the NA-OF Model

**Figure 2:**
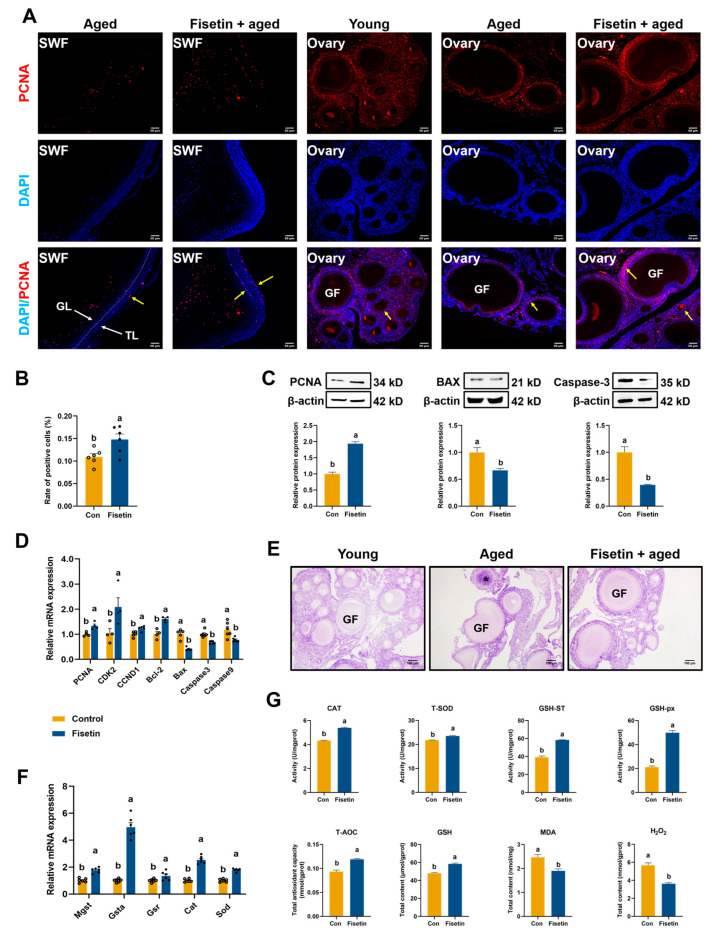
Effect of fisetin on proliferation and antioxidant enzymes in NA-OF model. (**A**) Typical images of PCNA expression (TRITC, red) in the ovary and SWF from young (280-day-old) and aged (580-day-old) laying chickens. TL: theca layer; GL: granulosa layer; GF: growing follicle. Yellow arrow: PCNA-positive cell. Nuclei were stained blue with DAPI. Scale bars: 50 μm and 100 μm. (**B**) PCNA positivity rate of SWF from aged laying chickens (*n* = 6). The proliferative level was determined by the PCNA positivity rate. (**C**) Western blotting showing the expression of PCNA, BAX, and Caspase-3 in SWF (*n* = 3). (**D**) The mRNA expression levels of cell cycle-related genes (*PCNA*, *CCND1*, *CDK2*, *Bcl-2*, *Bax*, *Caspase 3*, and *Caspase 9*) in SWF. (**E**) H&E staining ovaries harvested from young and aged laying chickens. Asterisk: atretic ovarian follicle. (**F**) Relative mRNA expression of antioxidant-related genes (*Mgst*, *Gsta*, *Gsr*, *Cat*, and *Sod*) in SWF (*n* = 6). (**G**) Levels of oxidative- and antioxidant-related parameters (T-SOD, CAT, GSH-px, GSH-ST, T-AOC, GSH, MDA, and H_2_O_2_) in SWF (*n* = 6). Values are shown as mean ± SEM. Different letters represent statistically significant differences among the groups (*p* < 0.05).

We further examined the effect of fisetin on cell proliferation in the ovaries of aged laying chickens. Immunofluorescence staining showed a significant decrease in the proliferative capacity of ovarian cells in aged chickens compared with young chickens (Figure 2A). However, the rate of PCNA positivity of SWF was significantly increased after fisetin treatment, compared with aged chicken in control diet (*p* < 0.05, Figure 2B). Additionally, fisetin increased PCNA (*p* < 0.001) expression and decreased BAX (*p* < 0.05) and Caspase-3 levels (*p* < 0.001), compared with the con group (Figure 2C). qRT-PCR analysis revealed that fisetin significantly increased the mRNA levels of *PCNA*, *CCND1*, *CDK2*, and *Bcl-2*, and downregulated *Bax*, *Caspase 3*, and *Caspase 9* (*p* < 0.05, Figure 2D) in aged SWF, compared with the con group. Representative images demonstrated that fisetin facilitated the development of growing follicles (Figure 2E).

As shown in Figure 2F, compared with the control, short-term fisetin intake enhanced antioxidant capacity, upregulating *Gsta*, *Cat*, *Mgst*, *Sod*, and *Gsr* expression in SWF (*p* < 0.05). Consistent with the gene expression data, levels of T-SOD, CAT, GSH-px, GSH-ST, T-AOC, and GSH increased, while MDA and H_2_O_2_ levels decreased (*p* < 0.05, Figure 2G). These findings suggest that fisetin effectively improves ovarian antioxidant capacity, providing preliminary evidence of its efficacy on chicken ovarian cells.

### 3.4. Fisetin Enhanced Ovarian Glucose Metabolism in the NA-OF Model

**Figure 3:**
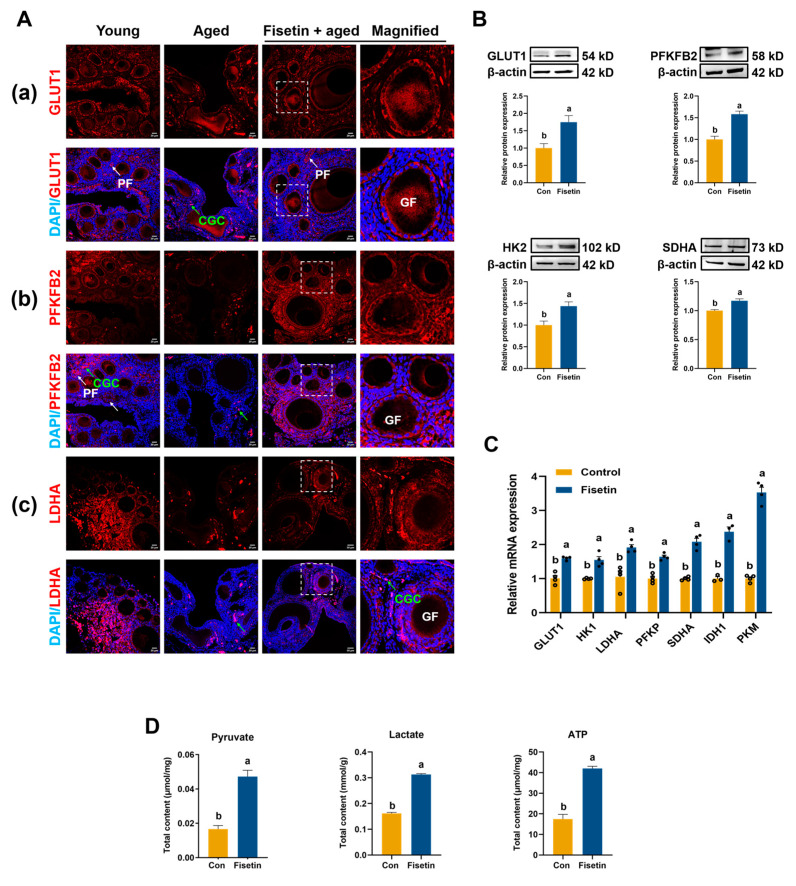
Effect of fisetin on ovarian glucose catabolism in the NA-OF model. (**A**) Immunofluorescence images of ovaries stained with GLUT1, PFKFB2, and LDHA antibodies (Red: positive cells; Blue: DAPI), and images in the white dotted boxes were enlarged on the right. Scale bar: 50 μm and 20 μm. Green arrow: cyst germ cell (CGC); white arrow: primordial follicle (PF); GF: growing follicle. (**B**) Relative protein expression of glucose metabolism-related enzymes (GLUT1, PFKFB2, HK2, SDHA) in SWF (*n* = 3). (**C**) Relative mRNA expressions of glycolysis-related genes (*GLUT1*, *HK1*, *LDHA*, *PFKP*, *SDHA*, *IDH1*, and *PKM*) in SWF. (**D**) Total contents of pyruvate, lactate, and ATP in SWF (*n* = 6). Values are shown as mean ± SEM. Different letters represent statistically significant differences among the groups (*p* < 0.05).

We next investigated glucose metabolism-related enzymes in aged ovaries. Immunofluorescence staining and confocal imaging revealed that GLUT1 was primarily expressed in the cytoplasm of granulosa cells (GC) of growing follicles, with minimal expression in stromal cells (Figure 3(Aa)). Compared with the young chickens, the expression of GLUT1 was significantly decreased in aged chickens and was predominantly expressed in cyst germ cells (CGC). However, fisetin treatment increased GLUT1 expression (Figure 3(Aa)). PFKFB2 was highly expressed in primordial follicles of young chicken ovaries, mainly expressed in CGC of aged chicken ovaries, while fisetin promoted its expression (Figure 3(Ab)). Additionally, LDHA was expressed in both the GC and CGC of young chickens, barely detected in the GC of aged chicken ovaries, and fisetin treatment promoted LDHA expression in CGC and restored its expression levels in stromal cells and GC (Figure 3(Ac)). Western blotting showed that fisetin-treated aged chickens had higher expression of GLUT1, PFKFB2, HK2, and SDHA, compared with the con group (*p* < 0.05, Figure 3B). Transcript levels of *GLUT1*, *HK1*, *LDHA*, *PFKP*, *SDHA*, *IDH1*, and *PKM* were also increased after fisetin treatment, compared with control (*p* < 0.01, Figure 3C). As products of glucose metabolism, the levels of ATP, pyruvate, and lactate in SWF were also upregulated by fisetin treatment (*p* < 0.001, Figure 3D).

### 3.5. Fisetin Alleviated Ovarian Injuries in the HA-OF Model

**Figure 4:**
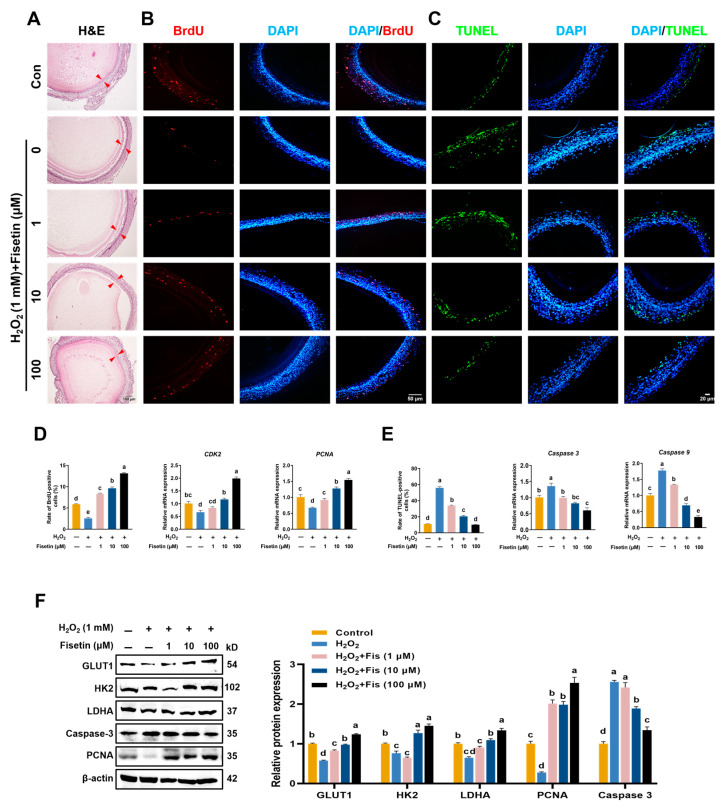
Effect of fisetin on the HA-OF model. (**A**) H&E staining images of SWF, scale bar: 100 μm. The red arrowhead indicates granulosa cell layer. (**B**) Immunofluorescence images of SWF stained with BrdU (red), scale bar: 50 μm. (**C**) TUNEL-stained (green) images of SWF, scale bar: 20 μm. (**D**) BrdU positivity rates and mRNA levels of cell cycle-related genes (*CDK2*, *PCNA*) in SWF (*n* = 4). (**E**) TUNEL positivity rates and relative expression levels of apoptosis-related genes (*Caspase 3*, *Caspase 9*) in SWF (*n* = 4). (**F**) Western blot analysis of GLUT1, HK2, LDHA, Caspase-3, and PCNA in SWF (*n* = 3). Values are presented as mean ± SEM. Different letters represent statistically significant differences among the groups (*p* < 0.05).

To investigate the mechanism of fisetin on the ovaries of aged laying chickens, we used an H_2_O_2_-induced aging ovarian follicle model (HA-OF) to simulate progressive ovarian degradation [21]. Histological examination with H&E staining showed that fisetin treatment significantly alleviated H_2_O_2_-induced ovarian follicle damage, allowing normal development (Figure 4A). Compared with the con and H_2_O_2_ group, fisetin also increased BrdU positivity and mRNA levels of *PCNA* and *CDK2*, indicating enhanced proliferation in a concentration-dependent manner (Figure 4B,D). Conversely, TUNEL staining confirmed significant apoptosis in the control and H_2_O_2_ groups, which was reduced by fisetin treatment (Figure 4C). Furthermore, the mRNA levels of *Caspase 3* and *Caspase 9* were decreased in fisetin-treated SWF compared with the H_2_O_2_ group (*p* < 0.001, Figure 4E). Western blot analysis demonstrated that fisetin (Fis) restored the protein expression of PCNA, GLUT1, HK2, and LDHA while reducing Caspase 3 expression compared with the H_2_O_2_ group (Figure 4F), with the most pronounced effect at 100 μM, indicating inhibition of H_2_O_2_-induced apoptosis.

### 3.6. Fisetin Restored Antioxidant Capacity and Glucose Metabolism in the HA-OF Model

**Figure 5:**
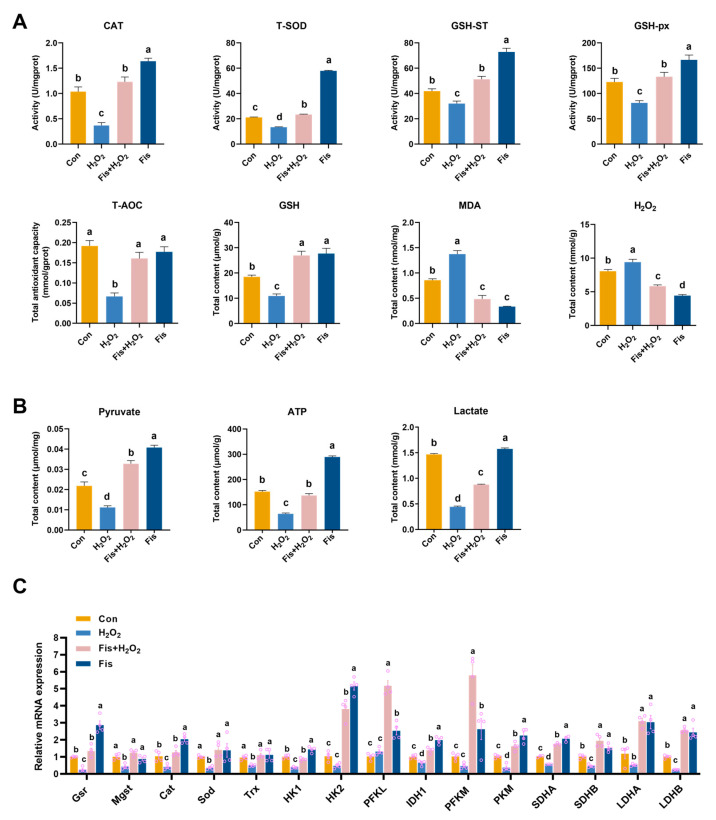
Effect of fisetin on antioxidant- and glucose metabolism-related enzymes in the HA-OF model. (**A**) Levels of oxidation- and antioxidant-related parameters (CAT, GSH-ST, GSH-px, T-SOD, T-AOC, GSH, MDA, and H_2_O_2_) in SWF (*n* = 6). (**B**) Levels of lactate, pyruvate, and ATP in SWF (*n* = 6). (**C**) Relative mRNA expression of antioxidant enzyme genes (*Gsr*, *Mgst*, *Cat*, *Sod*, and *Trx*) and glucose metabolism-related enzyme genes (*HK1*, *HK2*, *PFKL*, *IDH1*, *PFKM*, *PKM*, *SDHA*, *SDHB*, *LDHA*, and *LDHB*) in SWF (*n* = 4). Values are shown as mean ± SEM. Different letters represent statistically significant differences among the groups (*p* < 0.05).

Next, we investigated the effect of fisetin on antioxidant status and energy metabolism in the HA-OF model. H_2_O_2_-induced damage increased the expression of MDA and H_2_O_2_ in SWF compared with the H_2_O_2_ group, while this increase was reversed after fisetin treatment (*p* < 0.001, Figure 5A). Additionally, fisetin treatment elevated the activities of CAT, GSH-ST, GSH-px, T-SOD, and the levels of T-AOC and GSH, which are crucial for antioxidant defense compared with the H_2_O_2_ group (*p* < 0.01, Figure 5A). Furthermore, fisetin significantly upregulated the expression of antioxidant enzyme genes, including *Cat*, *Mgst*, *Sod*, *Gsr*, and *Trx* (Figure 5C). Fisetin also restored the levels of ATP, lactate, and pyruvate to near-normal levels, compared with H_2_O_2_ group (*p* < 0.01, Figure 5B) and increased the transcript levels of *HK1*, *HK2*, *PFKL*, *IDH1*, *PFKM*, *PKM*, *SDHA*, *SDHB*, *LDHA*, and *LDHB* (*p* < 0.05, Figure 5C).

### 3.7. Inhibition of NF-κB Signaling by Fisetin in the HA-OF Model

**Figure 6:**
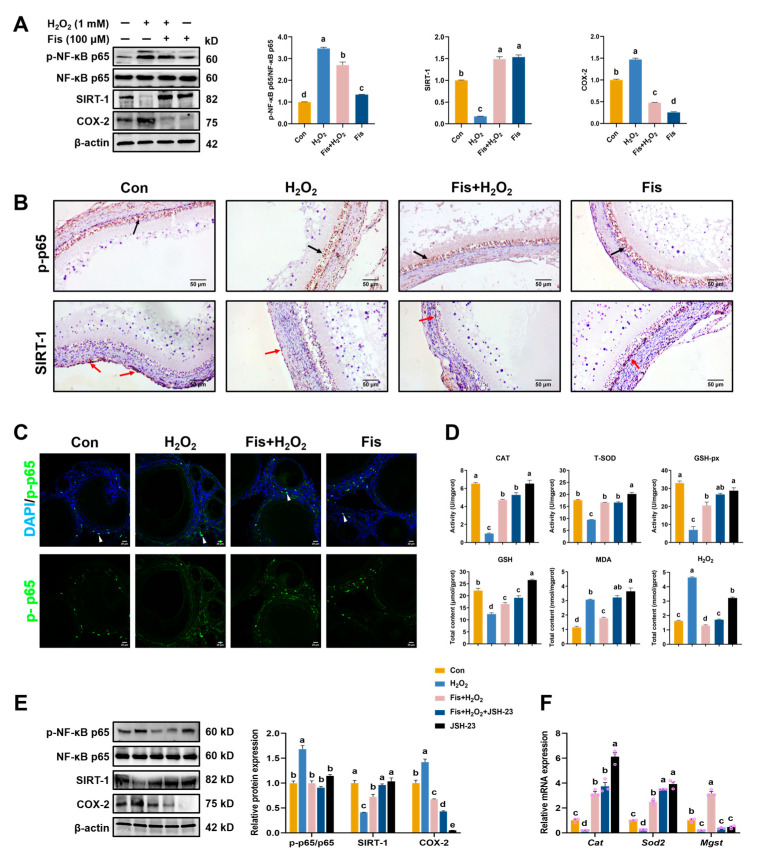
Effect of fisetin on the NF-κB signaling pathway in the HA-OF model. (**A**) Inhibition of NF-κB by fisetin in H_2_O_2_-induced SWF, as revealed by Western blot analysis (*n* = 3). Phosphorylated NF-κB p65, total NF-κB p65, SIRT-1, and COX-2 were analyzed. (**B**) Immunohistochemical staining for SIRT-1 and p-p65 in SWF. Black arrows indicate the localization of p-p65, primarily expressed in granulosa cells. Red arrows indicate the localization of SIRT-1, primarily expressed in theca cells. (**C**) Expression of p-p65 in ovaries (FITC, green). White arrows indicate the presence of phosphorylated p65 in ovarian follicles and its translocation to the nucleus. Scale bar: 20 μm. (**D**) Levels of oxidative and antioxidant-related parameters. (**E**) Western blot analysis of p-p65/p65, SIRT-1, and COX-2 in SWF (*n* = 3). (**F**) Levels of *Cat*, *Sod2*, and *Mgst* mRNAs in SWF (*n* = 3). Values are presented as mean ± SEM. Different letters represent statistically significant differences among the groups (*p* < 0.05).

We investigated the impact of fisetin on ovarian oxidative stress by examining NF-κB signaling in the HA-OF model. Fisetin treatment inhibited NF-κB phosphorylation without altering total NF-κB levels, and suppressed COX-2 expression (*p* < 0.001, Figure 6A), compared with the con and H_2_O_2_ group. Typically, NF-κB resides in the cytoplasm in an inactive state but becomes activated upon exposure to stimuli such as ROS, leading to its translocation to the nucleus for gene expression regulation. IHC staining analysis revealed nuclear translocation of p-p65 in numerous GC of SWF due to H_2_O_2_-induced damage, which was significantly mitigated by fisetin treatment (Figure 6B). SIRT-1, known for enhancing cellular resistance to oxidative stress, showed low expression in GC but was predominantly localized in the nuclei of the theca cells of SWF (Figure 6B). Compared with the control, H_2_O_2_ treatment reduced SIRT-1 expression (*p* < 0.001), which was reversed by fisetin supplementation, as confirmed by IHC staining and Western blotting (*p* < 0.001, Figure 6A,B). Immunostaining of ovarian sections indicated reduced p-p65 expression in the fisetin-treated group compared to the H_2_O_2_-treated group (Figure 6C), suggesting inhibition of NF-κB signaling by fisetin treatment.

We further explored whether fisetin inhibits ovarian oxidative stress via the NF-κB pathway by employing JSH-23, a selective NF-κB inhibitor, in the HA-OF model. JSH-23 treatment significantly increased mRNA levels of *Cat* and *Sod2*, while *Mgst* level remained unchanged in SWF, compared with the con and H_2_O_2_ groups (*p* < 0.01, Figure 6F). Consistently, the levels of CAT, T-SOD, GSH-px, and GSH were significantly elevated in the JSH-23 group, accompanied by decreased MDA and H_2_O_2_ levels, compared with H_2_O_2_ group (*p* < 0.05, Figure 6D). Additionally, JSH-23 significantly suppressed NF-κB phosphorylation and COX-2 expression while promoting SIRT-1 protein expression compared with the H_2_O_2_ group (*p* < 0.001, Figure 6E), which aligned with fisetin’s effects. These findings suggest that fisetin mitigates oxidative stress in SWF by inhibiting the NF-κB pathway and increasing SIRT-1 expression.

### 3.8. Activation of Fisetin on AKT and JNK Signaling in the HA-OF Model

**Figure 7:**
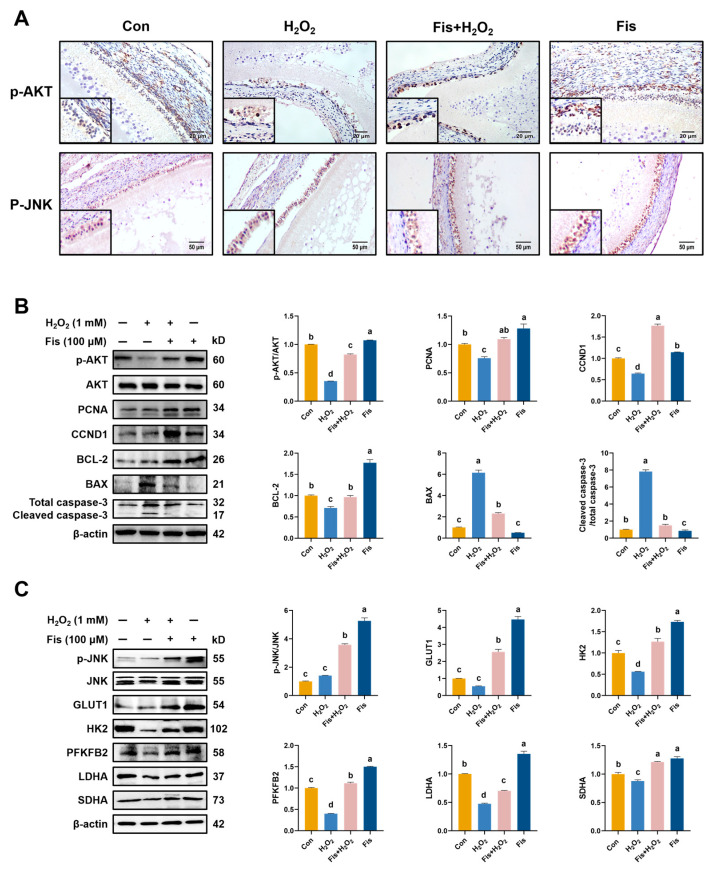
Effect of fisetin on the AKT signaling and the JNK signaling in the HA-OF model. (**A**) Immunohistochemical staining of p-JNK and p-AKT in SWF. Scale bars: 20 μm and 50 μm. (**B**) Activation of AKT signaling by fisetin in the H_2_O_2_-induced SWF. Phosphorylated AKT (Ser473) and total AKT were examined by Western blot. Quantification of AKT and its downstream signaling (p-AKT/AKT, PCNA, CCND1, BAX, BCL-2, and Caspase 3) was plotted on the right. The apoptotic level was determined by the normalized ratio of cleaved to total Caspase 3 and the protein quantification of BAX (*n* = 3). (**C**) Activation of JNK and regulation of glucose metabolism-related enzyme expression by fisetin, revealed by Western blot. Quantification of p-JNK/JNK, GLUT1, HK2, PFKFB2, LDHA, and SDHA is plotted on the right (*n* = 3). Values are shown as mean ± SEM. Different letters represent statistically significant differences among the groups (*p* < 0.05).

We investigated the potential influence of fisetin on AKT signaling, given its critical role in cell survival and energy metabolism. IHC staining and western blot data confirmed that fisetin actively facilitated the nuclear translocation of p-AKT in granulosa cells of SWF and enhanced p-AKT expression in the inner theca cells, compared with the H_2_O_2_ group (*p* < 0.001, Figure 7A,B). Additionally, the expression levels of PCNA, CCND1, and BCL-2, which are downstream targets of AKT, were significantly increased after fisetin treatment compared with the H_2_O_2_ group, while the protein levels of BAX and cleaved Caspase 3 were decreased (*p* < 0.001, Figure 7B). Furthermore, fisetin treatment promoted the nuclear translocation of p-JNK in granulosa cells of SWF and enhanced the levels of JNK phosphorylation, compared with the con and H_2_O_2_ group (*p* < 0.05, Figure 7A,C). Moreover, fisetin upregulated the protein levels of GLUT1, HK2, PFKFB2, LDHA, and SDHA compared with the H_2_O_2_ group (*p* < 0.001, Figure 7C), indicating its potential involvement in regulating glycolysis through JNK signaling.

### 3.9. Enhancement of Fisetin on JNK-Mediated Glucose Catabolism

**Figure 8:**
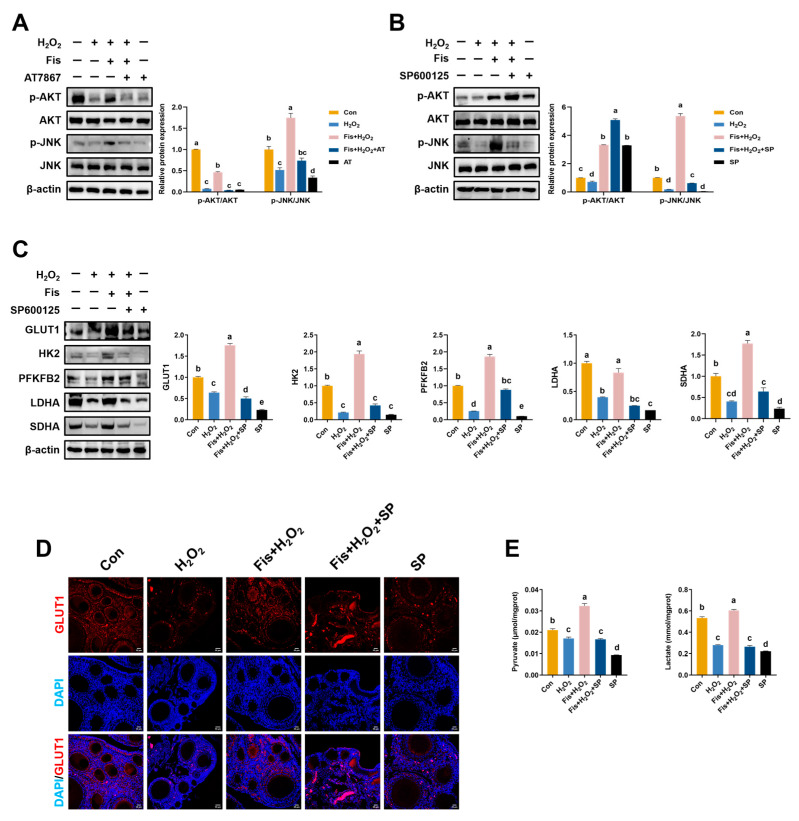
Fisetin promoted ovarian energy metabolism of laying chickens via AKT and JNK signaling pathways. (**A**) Activation of AKT and JNK signaling was inhibited by AT (100 μM). Total AKT, phosphorylated AKT, total JNK, and phosphorylated JNK were assessed in SWF by Western blot analysis. (**B**) Western blot and quantitative analyses of total AKT, phosphorylated AKT, total JNK and phosphorylated JNK expression in SWF (*n* = 3) after SP treatment (50 μM). (**C**) Western blot and quantitative analyses of GLUT1, HK2, PFKFB2, LDHA, and SDHA after SP treatment (50 μM) in SWF (*n* = 3). (**D**) Immunofluorescence images of ovaries stained with GLUT1 (TRITC, red). Scale bar: 20 μm. (**E**) Levels of lactate and pyruvate in SWF (*n* = 6). Values are shown as mean ± SEM. Different letters represent statistically significant differences among the groups (*p* < 0.05).

Given the crucial roles of AKT and JNK in follicular development, we treated the HA-OF model with the AKT inhibitor AT7867 (AT) and the JNK inhibitor SP600125 (SP), respectively. Compared with the con and Fis + H_2_O_2_ groups, AT treatment notably decreased p-AKT and p-JNK protein levels without affecting total protein levels (*p* < 0.01, Figure 8A). In contrast, SP inhibited p-JNK expression (*p* < 0.01), while having little impact on p-AKT levels, compared with the Fis + H_2_O_2_ group (*p* = 0.98, Figure 8B). In addition, SP significantly reversed the promoting effect of fisetin on the protein levels of GLUT1, HK2, PFKFB2, LDHA, and SDHA, compared with the Fis + H_2_O_2_ group (*p* < 0.001, Figure 8C). IF staining showed that SP treatment significantly inhibited the expression of GLUT1 in the ovary compared with the con and Fis + H_2_O_2_ groups (Figure 8D). Additionally, the production of pyruvate and lactate in SWF was significantly decreased by SP treatment, compared with the con and Fis + H_2_O_2_ groups (*p* < 0.001, Figure 8E).

### 3.10. Improvement of In Vitro Follicle Development, Antioxidant Properties, and Glucose Catabolism by Fisetin in the NA-OF Model

**Figure 9:**
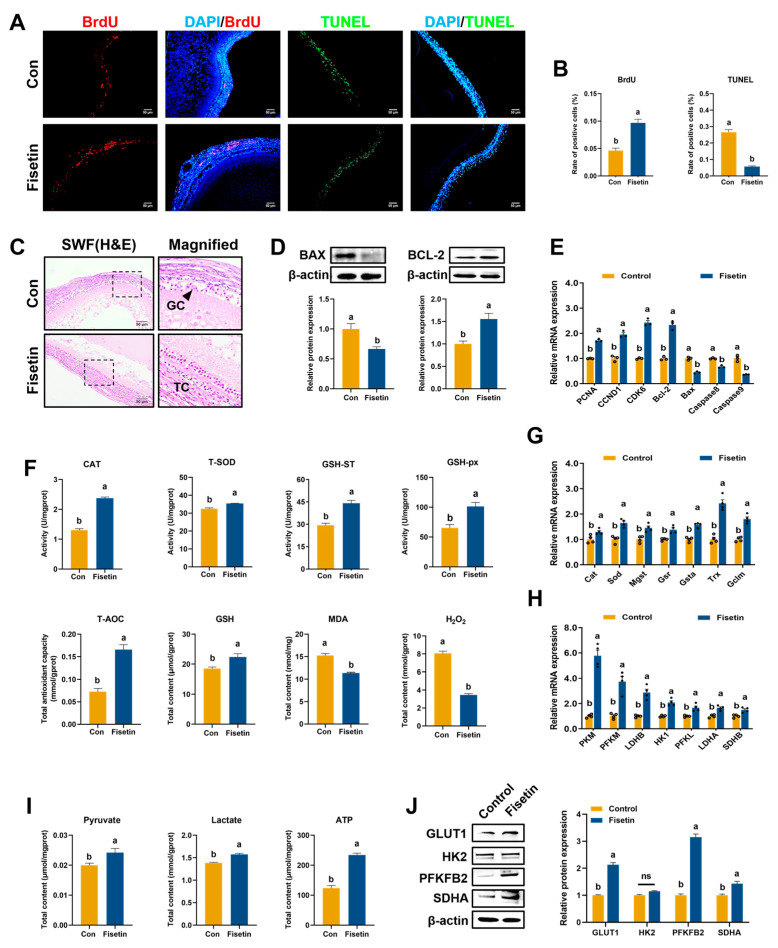
Effect of fisetin on the growth and development, antioxidant properties, and energy metabolism of aged SWF in vitro. (**A**) Representative images of BrdU (red) and TUNEL (green) assay of SWF in each group. The red dots represent proliferation-positive cells, and green dots represent apoptosis-positive cells. Scale bar: 50 μm. (**B**) Quantification of BrdU and TUNEL assay (*n* = 6). (**C**) Representative H&E staining images of SWF after 72 h culture in vitro, and images in the black dotted boxes were enlarged on the right. (**D**) Protein expression levels of BAX and BCL-2 in aged SWF (*n* = 3). (**E**) Relative mRNA levels of cell cycle-related genes (*PCNA*, *CCND1*, *CDK6*, *Bcl-2*, *Bax*, *Caspase 8*, and *Caspase 9*) in aged SWF (*n* = 3). (**F**) Levels of CAT, T-SOD, GSH-ST, GSH-Px, T-AOC, GSH, H_2_O_2_, and MDA in aged SWF (*n* = 6). (**G**) Relative mRNA levels of antioxidant-related genes (*Sod*, *Cat*, *Mgst*, *Gsr*, *Gsta*, *Trx*, and *Gclm*) in aged SWF (*n* = 4). (**H**) Transcription levels of glucose metabolism-related genes (*PKM*, *PFKM*, *LDHB*, *HK1*, *PFKL*, *LDHA*, and *SDHB*) in aged SWF (*n* = 4). (**I**) Contents of lactate, pyruvate and ATP in aged SWF (*n* = 6). (**J**) Relative protein levels of GLUT1, HK2, PFKFB2, and SDHA in aged SWF (*n* = 3). Values are shown as mean ± SEM. Different letters represent statistically significant differences among the groups (*p* < 0.05).

Finally, we examined the effects of fisetin on antioxidant status, energy metabolism, and growth in an in vitro NA-OF model. As shown in Figure 9A,B, fisetin treatment increased BrdU labeling and decreased TUNEL labeling in aged SWF after 72 h, compared with the control. H&E-stained images show more compact granulosa cell arrangement than control in aged SWF (Figure 9C). Fisetin also upregulated BCL-2 and downregulated BAX protein levels compared with the con group (*p* < 0.05, Figure 9D), alongside increased mRNA levels of *PCNA*, *CCND1*, *CDK6*, and *Bcl-2*, while reducing *Bax*, *Caspase 8*, and *Caspase 9* transcription (*p* < 0.05, Figure 9E). Fisetin improved antioxidant capacity in aged SWF, increasing the levels of CAT, T-SOD, GSH-ST, GSH-Px, GSH, and T-AOC while reducing H_2_O_2_ and MDA contents, compared with the con group (*p* < 0.05, Figure 9F). The expression levels of *Sod*, *Cat*, *Mgst*, *Gsr*, *Gsta*, *Trx*, and *Gclm* were higher in fisetin-treated SWF than the control (*p* < 0.05, Figure 9G). Additionally, fisetin significantly increased transcript levels of *PKM*, *PFKM*, *LDHB*, *HK1*, *PFKL*, *LDHA*, and *SDHB* (*p* < 0.05, Figure 9H). Western blotting revealed that the protein levels of GLUT1, PFKFB2, and SDHA were increased after in vitro culture (*p* < 0.05), and it had no significant effect on HK2 (*p* = 0.071), compared with the control (Figure 9J). Lastly, fisetin treatment improved ATP, lactate, and pyruvate production in SWF compared with the con group (*p* < 0.05, Figure 9I).

## 4. Discussion

In this study, we provide evidence that targeting the NF-κB, AKT, and JNK signaling pathways in ovarian follicles has the potential to be a promising strategy for ovarian aging treatment. Fisetin demonstrated superiority in terms of non-toxicity, energy metabolism restoration, and mitigation of oxidative stress by inhibiting ovarian NF-κB and its downstream signaling. In the NA-OF model, fisetin treatment rescued ovarian degeneration and follicular loss, restoring hormonal homeostasis. In the HA-OF model, fisetin repaired follicular damage, inhibited follicular cell apoptosis, and restored the antioxidant status and energy metabolism balance of follicles.

Reproductive aging is considered a natural phenomenon, but the decline in female ovarian function occurs earlier and faster than in any other organ in the body [1]. During this process, the number of follicles decreases, and the incidence of ovarian follicle atresia increases. In the poultry industry, aging not only reduces production but also disrupts calcium metabolism, leading to a decline in eggshell and bone quality [22]. However, extending the production period of laying hens faces challenges such as persistence and reduced egg quality. The production period of Hyline White laying chickens is approximately 80 weeks, after which egg production begins to decline more noticeably [14]. Our study demonstrated that fisetin supplementation in aged laying chickens effectively mitigated the decline in egg production and follicular atresia, and promoted follicular development, indicating significant improvement in follicle quantity and egg quality.

Estrogen is the primary hormone for maintaining the health of the female reproductive system. It plays a crucial role in improving cardiovascular health, maintaining oxidative balance, and reducing osteoporosis and cognitive impairment, while hypoestrogenism drives aging [23,24]. Similarly, during follicular development in poultry, reproductive hormones such as estrogen, follicle-stimulating hormone (FSH), and progesterone play a crucial role not only in regulating atresia but also in various physiological processes. These processes include stimulating cellular reproduction, promoting the development of primordial follicles, and facilitating yolk formation [25]. Studies have shown that 17β-estradiol is involved in the formation and activation of primordial follicles in chickens during early ovarian development [26]. In this study, fisetin supplementation effectively restored estrogen and progesterone levels in aged laying chickens, increasing progesterone levels by an average of about 55%. This may be achieved by upregulating the transcript levels of key rate-limiting enzymes for steroid hormone synthesis, specifically cytochrome P450 family members *CYP11A1* and *CYP19A1*. Previous research has shown that fisetin restored normal levels of estrogen and progesterone in letrozole-induced polycystic ovary syndrome [27], consistent with our findings.

Fisetin, a dietary flavonoid widely present in vegetables and fruits such as strawberries, apples, onions, and persimmons, has shown remarkable effects in antioxidant, anticancer, and neurodegenerative disease models [28,29]. Lipid peroxides and hydrogen peroxide serve as markers of oxidative stress, reflecting the harmful effects of ROS [30,31]. As end products of oxidation, MDA and H_2_O_2_ indicate the extent of tissue oxidative damage. Our findings from both in vivo and in vitro experiments demonstrated a notable decrease in MDA and H_2_O_2_ levels in the groups subjected to fisetin treatment. As an excellent antioxidant, fisetin prevents ovarian aging and enhances ovarian function by activating the expression of genes crucial for reducing oxidative damage. It significantly increased the mRNA expression of *Cat*, *Sod*, *Gsr*, and *Mgst*, as well as the activities of CAT, GSH-px, GSH-ST, and T-SOD, and the concentration of GSH. This treatment successfully restored the antioxidant status of aging ovarian tissues. Exogenous hydrogen peroxide often induces DNA damage and ROS, leading to ovarian dysfunction, including follicular atresia and oxidative stress [14,32]. In growing follicles, H_2_O_2_-induced toxicity leads to ovarian follicular apoptosis and activation of apoptotic signaling. Caspase 3 and caspase 9 transcript levels were upregulated, activated caspase 3 was highly expressed, and BAX expression was promoted. Notably, fisetin reversed this process through AKT-mediated inhibition of apoptosis. Additionally, fisetin dose-dependently promoted cell survival by increasing the expression of the anti-apoptotic protein BCL-2 and cell cycle regulators (PCNA, CCND1, and CDK2). In conclusion, these findings suggest that fisetin maintains normal ovarian follicular development by promoting cell proliferation, inhibiting apoptosis, and exerting antioxidant effects. The poultry industry faces numerous challenges, including high-density feeding, environmental stresses, and pathogen exposure, which can elevate oxidative stress levels. As a result, fisetin exhibits potential as a beneficial feed additive in addressing these issues.

In the context of poultry physiology, oxidative stress is commonly observed in the aging ovaries of chickens, leading to follicular atresia [33]. The complex interaction between oxidative stress and the aging ovaries highlights the need to elucidate the molecular mechanisms involved, with significant implications for reproductive health and fertility in poultry. NF-κB has emerged as a crucial protein in the cellular response to oxidative stress. Upon activation, it induces the expression of various proteins, including COX-2, iNOS, and TNF-α, triggering irreversible inflammatory damage [34]. Research has shown that fisetin can ameliorate lipopolysaccharide (LPS)-induced endometritis [35] and reduce airway inflammation [36]. Using Western blot analysis, we detected the presence of NF-κB p65 protein in prehierarchical follicles. Both H_2_O_2_-induced COX-2 protein expression and p65 phosphorylation were downregulated by fisetin. Previous studies have indicated that fisetin inhibits COX-2 expression by suppressing IκBα and p65 phosphorylation in LPS-induced septic acute kidney injury [37], which aligns with our results. Similarly, when ovarian follicles were treated with JSH-23, the NF-κB pathway and its downstream signaling were inhibited, and the antioxidant enzyme activities of ovarian follicles were significantly enhanced. These findings suggest that fisetin exerts a role similar to that of JSH-23 in alleviating oxidative stress in the ovaries of laying chickens by inhibiting the NF-κB signaling pathway.

As a member of the sirtuin family, SIRT1 plays crucial roles in various cellular processes, including aging, oxidative stress, energy metabolism, cell cycle regulation, and apoptosis [38]. Because SIRT1 functions as a regulator for numerous signaling pathways, abundant data indicate that activating upstream or downstream signals of SIRT1 reduces cell apoptosis, mitigates mitophagy defects, and upregulates antioxidant gene expression, thereby alleviating oxidative stress and follicular atresia [39,40]. Our previous study showed that pterostilbene restored the antioxidant capacity of D-galactose-induced SWFs by upregulating SIRT1/Nrf2 expression [41]. Numerous studies have demonstrated that SIRT1 regulates ovarian cell proliferation, endocrine activity, and inflammatory responses by controlling NF-κB through a negative feedback mechanism [42,43]. Our results demonstrated that fisetin facilitated SIRT1 expression and restored the reduction induced by H_2_O_2_. Consequently, fisetin is a promising SIRT1 activator and is emerging as a potential therapeutic agent for the treatment of age-related diseases.

Ovarian aging is often accompanied by a decline in energy metabolism [44]. This was confirmed by our findings, where the expression of GLUT1, PFKFB2, and LDHA was significantly downregulated in the ovaries of aged chickens compared to young chickens. A similar decrease in transcript and protein levels of enzymes involved in oxidative phosphorylation and glycolytic pathways was observed in the HA-OF model. In both in vivo and in vitro assays, fisetin treatment rescued the impaired energy metabolism of the ovary by upregulating the transcription and protein expression of key enzymes (GLUT1, HK2, PFKFB2, LDHA, and SDHA) in glucose catabolism, ultimately increasing the production of pyruvate, lactate, and ATP. A study has shown that administering fisetin orally to diabetic rats increases the activities of hexokinase and pyruvate kinase in the liver and kidney [45], aligning with our findings. One study confirmed that inhibition of glycolysis promoted the senescence of mouse oocytes, while in vitro supplementation of pyruvate and lactate prevented oocyte aging [46]. Previously, we found that JNK activation promoted glucose metabolism and cell proliferation in 60-month-old mouse spermatogonial stem cells [47]. Similarly, in H_2_O_2_-treated follicles, fisetin treatment significantly upregulated JNK phosphorylation levels. Protein expression of enzymes related to glucose metabolism and energy metabolites was also elevated in the aging follicle model. However, this promotion of glucose catabolism by fisetin was reversed by a JNK inhibitor. Although glucose catabolism in ovarian follicles involves mitochondrial oxidative phosphorylation, such as SDHA expression in the tricarboxylic acid cycle, we suggest that aged follicles are more dependent on glycolysis because HK2 and PFKFB2 mediate irreversible steps of glycolysis and are expressed at relatively high levels in the aging model. In conclusion, our results suggest that JNK activation promotes glucose metabolism in ovarian follicles of laying chickens.

AKT, a serine/threonine kinase, is crucial for cell survival and regulates various functions essential for maintaining energy homeostasis [48]. Our results revealed that fisetin actively facilitated AKT activation and downstream signaling in the HA-OF model of prehierarchical SWF. Consequently, the expression of cell development factors such as PCNA, CCND1, and CDK2, which are downstream targets of AKT, was upregulated. Given the roles of AKT and JNK in the senescent follicle model, we also explored the relationship between AKT and JNK in fisetin-mediated regulation of ovarian senescence. The results showed that AKT acts as an upstream regulator of JNK, influencing phosphorylation events in the JNK pathway. However, the limitations of the experiments suggest that the specific interactions between AKT and JNK need further exploration. In conclusion, our findings highlight the importance of the cascade activation of AKT and JNK in regulating ovarian energy metabolism in laying chickens.

## 5. Conclusions

**Figure 10:**
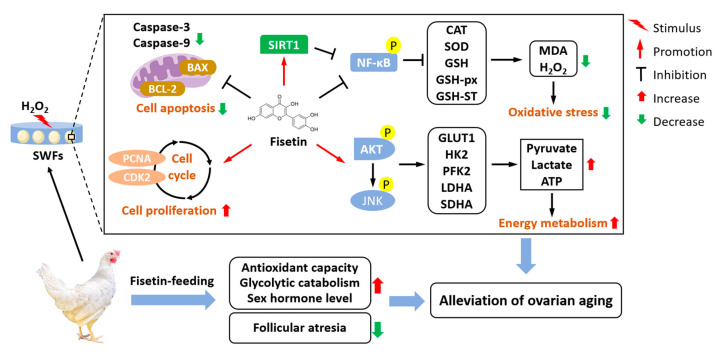
Schematic diagram illustrating the mechanism by which fisetin alleviates ovarian aging in laying chickens.

Dietary fisetin supplementation improved egg production, restored ovarian antioxidant capacity and energy metabolism in aged laying chickens. Furthermore, fisetin offers a potential strategy for optimizing ovarian energy supply through activation of the AKT and JNK signaling pathways. In conclusion, fisetin has multiple benefits as a feed additive, supporting both the health and reproductive performance of poultry (Figure 10).

## Figures and Tables

**Table 1:** Effect of fisetin on laying performance of aged laying chickens.

Item	Con	Fis	SEM	*p*-Value
Average egg weight, g	61.80 ^b^	67.83 ^a^	1.589	0.0035
Egg production, %	67.95 ^b^	78.43 ^a^	0.030	0.0036
Eggshell strength, kgf	2.462 ^b^	3.973 ^a^	0.440	0.0063
Eggshell thickness, mm	0.335 ^b^	0.3917 ^a^	0.013	0.0016
Egg yolk color	7.167 ^a^	6.167 ^b^	0.236	0.0017
Albumen height, mm	6.933	6.633	0.306	0.3498
Haugh unit	81.90	79.17	1.890	0.1786

Con group = basal diet; Fis group = basal diet + 50 mg/kg fisetin powder. Values are represented as mean and SEM, *n* = 6. ^a,b^ Means within a row with different superscripts differ significantly (*p* < 0.05).

## Data Availability

All data analyzed are contained within the article.

## Supplementary Materials

The following supporting information can be downloaded at: https://www.mdpi.com/2076-3921/13/12/1432/s1, Table S1: Primary antibodies used in the study; Table S2: Primers used for quantitative RT-PCR.
