# Supplementary material for: Flavonoid Fisetin Alleviates Ovarian Aging of Laying Chickens by Enhancing Antioxidant Capacity and Glucose Metabolic Homeostasis
Source: Antioxidants (Basel). 2024 Nov 21;13(12):1432. doi: 10.3390/antiox13121432 (PMC11672761; doi:10.3390/antiox13121432)
Supplement: Supplementary file 1 [file antioxidants-13-01432-s001.zip › antioxidants-3287841-supplementary.pdf]

Supplementary data

Table S1: Primary antibodies used in the study.

Primary antibodies	Manufacturer and Product code	Dilution	Source
AKT	ABclonal (A17909)	1:1000	Rabbit
p-AKT	ABclonal (AP0098)	1:1000	Rabbit
caspase 3	ABclonal (A16794)	1:1000	Rabbit
JNK	ABclonal (A2462)	1:1000	Rabbit
p-JNK	ABclonal (AP1337)	1:1000	Rabbit
HK2	ABclonal (A0994)	1:1000	Rabbit
COX-2	Boster Bio (BA0738)	1:1000	Rabbit
BAX	HUABIO (ET1603-34)	1:1000	Rabbit
BCL-2	HUABIO (ER0602)	1:2000	Rabbit
CCND1	HUABIO (ET1601-31)	1:2000	Rabbit
PCNA	HUABIO (ET1605-38)	1:2000	Rabbit
NF-κB p65	HUABIO (ER0815)	1:1000	Rabbit
p-NF-κB p65	HUABIO (ET1604-27)	1:1000	Rabbit
SIRT-1	HUABIO (M1506-3)	1:500	Mouse
LDHA	HUABIO (ER00702)	1:2000	Rabbit
PFKFB2	HUABIO (ER1915-04)	1:1000	Rabbit
SDHA	HUABIO (ET1703-40)	1:500	Rabbit
GLUT1	HUABIO (ER1510-11)	1:500	Rabbit
CYP11A1	HUABIO (ER1906-98)	1:500	Rabbit
β-actin	HUABIO (EM21002)	1:50000	Mouse

AKT = protein kinase B; p-AKT = phosphorylated protein kinase B; Bcl-2 = B-cell lymphoma-2; BAX = Bcl-2-associated X protein; caspase 3 = cysteine-aspartic acid protease 3;

CCND1 = cyclin D1; COX-2 = cyclooxygenase-2; CYP11A1 = cytochrome P450, family 11, subfamily A, polypeptide 1; GLUT1 = glucose transporter 1; HK2 = hexokinase 2; JNK = c-Jun N-terminal kinase; p-JNK = phosphorylated c-Jun N-terminal kinase; LDHA = lactate dehydrogenase A; NF-κB p65 = nuclear factor kappa B p65; p-NF-κB p65 = phosphorylated NF-κB p65; PCNA = proliferating cell nuclear antigen; PFKFB2 = 6-phosphofructo-2-kinase/fructose-2,6-bisphosphatase 2; SDHA = succinate dehydrogenase complex, subunit A; SIRT-1 = silent information regulator 1;β-actin = beta-actin.

Table S2: Primers used for quantitative RT-PCR

Gene	Accession number	Primer sequence (5′-3′)	Product size (bp)
Bax	XM_015290060.2	F: GGATGACAGGAAAGTACGGCA<br>R: TCACCAGGAAGACAGCGTAT	173
Bcl-2	NM_205339.2	F: ATCGTCGCCTTCTTCGAGTT<br>R: ATCCCATCCTCCGTTGTCCT	150
Caspase3	NM_204725.1	F: CAGCTGAAGGCTCCTGGTTT<br>R: GCCACTCTGCGATTTACACG	98
Caspase8	NM_204592.4	F: TAAAATGACCAGCCGACCCC<br>R: TCTGCATCCACATGTGTCCC	126
Caspase9	XM_424580.6	F: GCTTGTCCATCCCAGTCCAA<br>R: TGCTGCTGACACCTTCACCATTC	95
Cat	NM_001031215.2	F: TCAGGAGATGTGCAGCGTTT<br>R: TCTTACACAGCCTTTGGCGT	109
CDK2	NM_001199857.1	F: TCCGTATCTTCCGCACGTTG<br>R: GCTTGTTGGGATCGTAGTGC	183
CDK6	NM_001007892	F: GCACGCCGGGATAATACAGA<br>R: CATGAGTACCAAAGCCCCGT	234
CYP11A1	NM_001001756.2	F: GGTGGCATACCGTGACTACC<br>R: ACAAAGTCCTGGCTCACCTG	159
CYP19A1	NM_001001761	F: CCTCTGCTGGAGATGGTTTT<br>R: GCTGATCCACTTTAGTCACTCTGA	68
Gclm	NM_001007953.1	F: CCATAGGCACCTCTGACCTTG<br>R: CGGCATCACGCAACATGAAG	110
Glut1	NM_205209.1	F: GCAAGATGACAGCTCGCCT<br>R: GTCTTCAATCACCTTCTGCGG	110
Gsr	XM_015276627.1	F: TCCTGACTACGGCTTCGAGA<br>R: AACTTGCCGTAACCACGGAT	150
Gsta	NM_204818.2	F: GCAGAGCCATCCTCAGCTAC<br>R: CCTTTGCCTCAGGTGGAGAG	150
HK1	NM_204101.1	F: GGAGGATCAGGTCAAAAAGATCG<br>R: TTCACTGTCGCTGTTGGGTT	141
HK2	NM_204212.1	F: GAAGGGTTTCAAAGCCACGG<br>R: AGGTCAAACTCCTCTCTGCG	87
IDH1	XM_004942682.3	F: GGAACAGTCTTCAGGGAGGC<br>R: CGTAAGCATGACGGCCAATG	94
LDHA	NM_205284.1	F: GAAGACGCCGGCAGTACA<br>R: ACCAACCACGCTGATCTTGT	101
LDHB	NM_204177.2	F: ACGTTATGGCGACCCTGAAG<br>R: ATCACAAAGACCCTTGCCGA	143
Mgst	NM_204794.2	F: AAAGAACAGAAACCCCATTCAG<br>R: GCACAGAGGGACATTTTGATT	82
PFKL	NM_001318442.1	F: CCTCTGAGGGCAAGGGAATC<br>R: CTTGGTCCCATAGTTGCGGT	101
PFKM	NM_204223.2	F: AGCAGACAGACTTCCAGCAC<br>R: GGGATTTTGGAGGGGTCGTT	198
PFKP	XM_046911664.1	F: GGAGCGGTCGGAGGAAAC<br>R: CTCGAAGAACTTGGGCTGGT	165
PKM	XM_015278796.2	F: CATGCAGCACGCTATTGCTC<br>R: GTGGTGTACACTGTGGCGTA	88
SDHA	NM_001277398.1	F: ATTCCCGTTTTGCCTACGGT<br>R: GGGAGTTTGCTCCAAGACGA	172
SDHB	NM_001080875.3	F: ATTGCTGGTGGAAACACCCT<br>R: TCCTTGCCCTGTTTCGACTC	191

Sod	NM_205064.1	F: GGCAATGTGACTGCAAAGGG<br>R: CCCCTCTACCCAGGTCATCA	133
Sod2	NM_204211.1	F: TCCTGACCTGCCTTACGACT<br>R: TGCCAGCGCCTCTTTTGTATT	139
Trx	NM_205453.1	F: GTGCATGCCAACATTCCAGT<br>R: CTCCATGGCGGGAGATTAGAC	118
β-actin	NM_205518.2	F: CCAGCCATGTATGTAGCCATCCAG<br>R: GGTAACACCATCACCAGAGTCCATC	93

Bcl-2 = B-cell lymphoma-2; *Bax* = Bcl-2-associated X protein; *Caspase 3* = cysteine-aspartic acid protease 3; *Caspase 8* = cysteine-aspartic acid protease 8; *Caspase 9* = cysteine-aspartic acid protease 9; *Cat* = catalase; *CCND1* = cyclin D1; *CDK2* = cyclin-dependent kinase 2; *CDK6* = cyclin-dependent kinase 6; *CYP11A1* = cytochrome P450, family 11, subfamily A, polypeptide 1; *CYP19A1* = cytochrome P450, family 19, subfamily A, polypeptide 1; *Gclm* = glutamate-cysteine ligase modifier subunit; *Glut1* = glucose transporter 1; *Gsr* = glutathione-disulfide reductase; *Gsta* = glutathione S-transferase alpha; *HK1* = hexokinase 1; *HK2* = hexokinase 2; *IDH1* = isocitrate dehydrogenase 1; *LDHA* = lactate dehydrogenase A; *LDHB* = lactate dehydrogenase B; *Mgst* = microsomal glutathione S-transferase; *PCNA* = proliferating cell nuclear antigen; *PFKL* = phosphofructokinase liver type; *PFKM* = phosphofructokinase muscle type; *PFKP* = phosphofructokinase platelet type; *PKM* = pyruvate kinase M; *SDHA* = succinate dehydrogenase complex flavoprotein subunit A; *SDHB* = succinate dehydrogenase complex flavoprotein subunit B; *Sod* = superoxide dismutase; *Trx* = thioredoxin.
